# Functional significance of erythropoietin in renal cell carcinoma

**DOI:** 10.1186/1471-2407-13-14

**Published:** 2013-01-10

**Authors:** Christudas Morais, David W Johnson, David A Vesey, Glenda C Gobe

**Affiliations:** 1 Centre for Kidney Disease Research, School of Medicine, University of Queensland at Princess Alexandra Hospital, Building 33, Brisbane, Queensland, 4102, Australia; 2 Department of Renal Medicine, The University of Queensland at Princess Alexandra Hospital, Brisbane, Queensland, 4102, Australia

## Abstract

One of the molecules regulated by the transcription factor, hypoxia inducible factor (HIF), is the hypoxia-responsive hematopoietic factor, erythropoietin (EPO). This may have relevance to the development of renal cell carcinoma (RCC), where mutations of the von Hippel-Lindau (VHL) gene are major risk factors for the development of familial and sporadic RCC. VHL mutations up-regulate and stabilize HIF, which in turn activates many downstream molecules, including EPO, that are known to promote angiogenesis, drug resistance, proliferation and progression of solid tumours. HIFs typically respond to hypoxic cellular environment. While the hypoxic microenvironment plays a critical role in the development and progression of tumours in general, it is of special significance in the case of RCC because of the link between VHL, HIF and EPO. EPO and its receptor, EPOR, are expressed in many cancers, including RCC. This limits the use of recombinant human EPO (rhEPO) to treat anaemia in cancer patients, because the rhEPO may be stimulatory to the cancer. EPO may also stimulate epithelial-mesenchymal transition (EMT) in RCC, and pathological EMT has a key role in cancer progression. In this mini review, we summarize the current knowledge of the role of EPO in RCC. The available data, either for or against the use of EPO in RCC patients, are equivocal and insufficient to draw a definitive conclusion.

## Background

Renal cell carcinoma (RCC) accounts for 3% of all adult cancers, and 90-95% of neoplasms of the kidney. It is a highly heterogeneous disease with many distinct histologic subtypes [1,2]. Clear cell RCC, arising from the proximal tubular epithelial cells (PTEC) is the most common sporadic subtype constituting 70-80% of RCC, followed by papillary (10-15%) and chromophobe (5%) RCC [3]. RCC can be either familial or sporadic. Both forms are often associated with distinct genetic mutations, of which the most prominent are the von Hippel-Lindau (VHL) gene mutations. The VHL syndrome, which is the result of a germ line mutation in the VHL gene, is the major predisposing factor for familial RCC [4-7]. In sporadic RCC, biallelic inactivation of the VHL gene, either through hyper-methylation or mutation, is the predominant risk factor. The VHL gene is hyper-methylated in about 19% and mutated in 34-56% of sporadic clear cell RCC [5,8-13]. Clear cell RCC is the leading cause of death in patients with VHL mutations [14]. Despite the recent advancements in the management of RCC patients, death rates have remained unchanged [15,16]

### The VHL-HIF-EPO pathway

**Figure 1:**
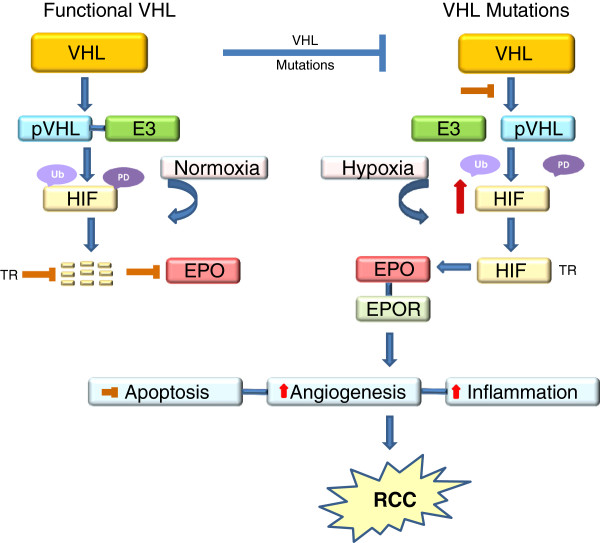
**The putative role of VHL-HIF-EPO pathway in RCC progression. **A functional VHL gene produces pVHL, which forms a pVHL-E3 ligase complex and mediates the poly ubiquitination (Ub) and proteasomal degradation (PD) of HIF. As a result, the translocation (TR) of HIF to the nucleus and the subsequent transactivation of HIF regulated molecules, including EPO is prevented. When the VHL gene is mutated, the production of pVHL and the formation of the pVHL-E3 ligase complex are either impaired or prevented. Subsequently, HIF is stabilized and up-regulated, and translocated to the nucleus, where it dimerizes with other HIF subunits and transactivates HIF responsive genes including EPO. EPO binds to its receptor EPOR and mediates some of the biological aspects of cancer progression such as increase in angiogenesis and inflammation and decrease in intrinsic and drug-induced apoptosis. Apart from VHL mutations, hypoxia is the single major factor that regulates the production of EPO. In normoxic conditions, the HIF is degraded, whereas in hypoxia, HIF is stabilized and lead leads to the activation of EPO.

As the tumour microenvironment is often hypoxic, tumour cells undergo adaptive changes to facilitate their survival. One such survival mechanism under hypoxic conditions is the up-regulation of the transcription factor hypoxia inducible factor (HIF). HIF has two subunits, HIF-α (which has three further subunits HIF-1α, HIF-2α and HIF-3α) and HIF-β [17,18]. While both subunits are constitutively expressed, the tissue levels of HIF-α, unlike HIF-β, are determined by the intracellular oxygen tension. Under normoxic conditions, HIF-α is rapidly degraded, an event largely mediated by a functional VHL [19-23]. The functional protein of VHL, pVHL, forms complexes with elongin B, elongin C, Rbx1 and cullin 2 to form a pVHL- E3 ubiquitin ligase complex (pVHL-E3 complex) [24-27]. The pVHL-E3 complex then binds to HIF-α, leading to its polyubiquitination and proteasomal degradation [25,28-32] (Figure 1). In the absence of a functional pVHL, secondary to VHL mutations, the formation of the pVHL-E3 complex and its binding to HIF-α are inhibited and therefore, the degradation of HIF-α is prevented even in normoxic conditions [23]. This leads to the stabilization and accumulation of HIF-α in cells. As a result, HIF-α is translocated to the nucleus, where it dimerizes with HIF-β, binds to hypoxia-responsive elements of the DNA and transactivates many downstream hypoxia-inducible molecules that are known to promote angiogenesis, proliferation, drug resistance and tumour progression [6,7,23,25,28] (Figure 1).

One such hypoxia-inducible molecule is the glycoprotein hormone erythropoietin (EPO). Apart from inducing EPO production through HIF, VHL mutations can directly up-regulate EPO without HIF activation [33,34]. Although clear cell RCC is thought to arise from the PTEC, normal PTEC do not express detectable levels of EPO even under hypoxic conditions [35-37]. Therefore, it is believed that VHL mutations play a key role in transforming a non-EPO expressing PTEC into an EPO-producing RCC [35-37]. While the hypoxic trigger of EPO is a major problem in cancer biology in general, this is of special significance in the case of RCC, because of the direct regulation of EPO by HIF. EPO is the only hematopoietic growth factor whose production is regulated by local hypoxia [38]. If that is the case, EPO is more likely to be a local player in cancer progression, rather than contributor of metastatic progression.

### EPO

The liver is the major site of EPO production in the foetus. At birth, there is a liver to kidney switch and, in adults, the peritubular fibroblasts of the renal cortex are the major sites of EPO production [39-45]. The hepatocytes and perisinusoidal Ito cells of the liver (hepatic stellate cells known for storage of vitamin A) are the major extrarenal sites of EPO production [43-45]. Other than the kidneys and the liver, EPO and EPOR are expressed in various non-hematopoietic tissues, such as vascular endothelial cells, the uterus, central nervous system and solid tumours [46]. While EPO is the essential hematopoietic growth factor for erythropoiesis in hematopoietic tissues, in non-hematopoietic tissues, and especially tumours, it inhibits apoptosis, stimulates angiogenesis, promotes drug resistance and increases cell proliferation [47-50]. The biological or oncogenic effects of EPO are mediated through interactions with its receptor, EPOR [51]. The EPO/EPOR interaction activates the cytoplasmic tyrosine kinase, Janus kinase 2 (JAK2), which in turn phosphorylates several cytoplasmic tyrosine residues in the cytoplasmic tail of Epo-R [41,52-55]. The phosphorylated cytoplasmic tail of EPOR acts as a docking site for proteins that contain Src-homology 2 (SH2) domains, for example STAT1, STAT3 and STAT5a/b, and initiates a cascade of signalling pathways that either promote erythropoiesis or tumour progression, depending on the target site [41,52-55].

Two important issues remain to be elucidated. First, it is not clear whether or not there is a difference in the production of EPO between RCC with a normal VHL and a mutated VHL. Second, irrespective of any difference in production, it is not clear whether or not there is a difference between the biological activity of EPO produced by a VHL wild type and a VHL mutant RCC.

### EPO and EPOR expression in RCC

Many studies have reported the over expression of EPO and EPOR in human RCC (Table 1) especially clear cell RCC [11,50,56-71]. This is because of the high rate of VHL mutations, and the subsequent overproduction and stabilization of HIF in clear cell RCC compared with any other subtypes [37]. RCC cells isolated from patients also express EPO and EPOR in culture [72-79], although conflicting findings have also been reported [37,58]. One unresolved issue is the correlation between EPO/EPOR expression and prognosis. With one exception [50], all studies to date [11,56-62] (Table 1) have failed to find an association between EPO/EPOR expression and survival. Despite the frequent expression of EPO and EPOR in RCC, approximately 35% of RCC patients develop anaemia, whilst only 1-5% experience paraneoplastic polycythaemia [37,47,62,80-83]. Possible explanations for this seemingly paradoxical finding in the face of elevated EPO blood levels include tumour-induced EPO inactivity (or reduced activity), EPO hyporesponsiveness, iron deficiency and inflammation.

**Table 1:** Expression of EPO and EPOR in RCC*

**Samples**	**Parameters**	**Method**	**Number of samples**	**% expression**	**References**
Serum	EPO	ELISA	165	33	[57]
Serum	EPO	ELISA	49	8	[58]
Tissues	EPO	IHC^#^	19	52	[59]
Tissues	EPO & EPOR	IHC	11	100	[60]
Tissues	EPO	IHC	20	100	[61]
Tissues	EPO	IHC	113	33	[50]
Tissues	EPO	IHC	82	88	[11,56]
Serum	EPO & EPOR	IHC	195	83 (tissue EPO)	[62]
Tissues		ELISA		33 (serum EPO)?	
				56 (tissue EPOR)	

*Apart from the publications that are listed in the table, there are many case reports involving one or two patients [63-71].

^#^IHC, immunohistochemistry.

### Does the EPO/EPOR pathway have functional significance in RCC?

Because EPO and EPOR are expressed in RCC (and in other cancers [46]), the use of recombinant human EPO (rhEPO) to treat anaemia in cancer patients has been subject to considerable debate. It is argued that the binding of exogenous rhEPO with EPOR might attenuate tumour growth by decreasing hypoxia (through erythrocyotosis), and thereby HIF, and the subsequent expression of downstream molecules that facilitate angiogenesis and other features of cancer progression [46]. The alternative argument is that binding of exogenous rhEPO with EPOR might, theoretically, initiate autocrine/paracrine effects that will promote tumour progression through inhibiting apoptosis, accelerating proliferation, promoting angiogenesis and enhancing drug resistance [46]. There are data available to support both views.

#### Beneficial effects of EPO in RCC

Immunotherapy with interleukin-2 (IL-2), which offers a short term response in 10-15% of RCC patients, is routinely used in the management of metastatic RCC. A high circulating level of vascular endothelial growth factor (VEGF) has been shown to predict IL-2 resistance in patients with metastatic RCC [84]. As hypoxia is one of the stimulators of VEGF, the correction of anaemia (or anaemia-induced hypoxia) with EPO would counteract the pro-angiogenic actions of VEGF and reverse IL-2 resistance [85]. Based on these assumptions, in a Phase II trial, Lissoni and colleagues [85] treated metastatic RCC patients, who had already been on IL-2, with a combination of IL-2 and EPO (10,000 units, 3 times a week). Apart from counteracting VEGF-related IL-2 resistance, EPO controlled cancer growth and reduced the toxicity of IL-2. A case report by Rubins [69] shows that treatment with EPO of a large volume metastatic RCC, which was refractory to immunotherapy, resulted in complete remission of all metastatic lesions. A French study that treated 20 patients with subcutaneous EPO for metastatic RCC demonstrated a complete response in one, partial response in three and disease stabilization in ten patients [86]. Janik and colleagues [87] reported that two polycythemic patients with EPO-producing RCC obtained partial or complete response to a combination of IL-2 and interferon-α treatment, suggesting that EPO-producing RCC may be an indicator of immunotherapy response. Carvalho and colleagues [88] reported that concomitant treatment with EPO enhanced the cytotoxicity of vinblastine and daunorubicin in RCC cell lines. Furthermore primary cultures of RCC transfected with erythropoietin-cDNA were more susceptible to lysis by lymphokine-activated killer cells [89].

#### Adverse effects of EPO in RCC

To the best of our knowledge, there are two reports that show adverse effects of EPO in RCC patients. In a case report, Sungur [70] describes of a patient who developed local recurrence of RCC while on EPO treatment. The patient had a left radical nephrectomy for RCC and the disease recurred 2 years later in the right kidney, for which a partial nephrectomy was performed. Subsequently, the patient received hemodialysis three times per week along with EPO, 12000U/week, for the first 6 weeks and then a maintenance dose of 4000U/week for 1 year [70]. Fourteen months later, ultrasonography showed a recurrent tumour in the adrenal gland, which was cured by right adrenalectomy. Interestingly through, the patient continued on EPO (4000 u/WK) and remained tumour-free for more than 9 months. Given the case history, it is difficult to conclude whether EPO was the cause of the recurrent tumour. Apart from this report, in the French study mentioned above [86], the remaining six of the 20 patients displayed progressive disease in response to EPO. *In vitro* studies from our laboratory showed that RCC cells treated with EPO developed resistance to cisplatin treatment [49].

Although not in RCC, it is worth mentioning the adverse effects of EPO administration in other cancers, especially breast cancer and head and neck cancer. In breast cancer, a phase III study on the use of EPO was stopped because of increased mortality, tumour progression and increased incidence of thrombotic and vascular events [90]. In a double-blind, placebo-controlled study, Henke and colleagues reported a poorer outcome for head and neck cancer patients who were treated with EPO [91]. These studies prompted the FDA to issue a black box warning on the use of EPO or erythroid-stimulating agents in cancer patients [92]. A review by Hadland and Longmore details the potential dangers of erythroid-stimulating agents in cancer therapy [93].

None of the clinical trials has explored the molecular mechanism of the EPO-mediated adverse events. While such mechanisms will undoubtedly be multifactorial, one common pathway by which HIF and EPO could potentially enhance cancer progression is by phosphatidylinositol3-kinase/Protein kinase B/mammalian target of rapamycin (PI3K/Akt/mTOR)–mediated EMT. This is best known in head and neck cancer but may well apply to RCC as well. HIF plays a crucial role in EMT of cancer cells and the PI3K/Akt/mTOR pathway plays a central role in this process. Both HIF and EPO activate this pathway. Phosphorylation of PI3K leads to the activation of Akt, which in turn activates mTOR [94,95]. This can be executed directly by HIF *per se* or through one of the many pro-inflammatory cytokines that are up-regulated in cancer patients, for example tumour necrosis factor-α [94-97]. To support this view, two recent studies have shown that hypoxia induced-EPO [98] and exogenous rhEPO [99] activate the PI3K/Akt/mTOR in retinal, and head and neck cancer cells respectively.

#### Neutral effects of EPO in RCC

There is at least one study that shows a neutral effect of EPO in cultured RCC cell lines. Treatment of 22 different cell lines, including 2 RCC cell lines, with rhEPO (dose range 0.01-100 U/ml) did not induce any significant changes in clonal growth or proliferation. Furthermore, a neutralizing anti-human EPO antibody had no effect on the clonal growth of these RCC cell lines thereby ruling out any autocrine effects of EPO [100].

### Conclusions and future directions

EPO is of special interest in RCC because of its direct regulation by the VHL-HIF pathway. As rhEPO is widely used in clinical practice for the treatment of anaemia associated with various disorders including cancer, the expression of EPO and EPOR in the kidney and especially in RCC has been a cause for concern. There are two schools of thought. One argues that exogenous rhEPO would correct hypoxia by increasing oxygenation, and therefore, would prevent or stabilize cancer progression. The other school argues that the binding of rhEPO with EPOR would enhance the progression of cancer. While each view has its own merit, a review of the available information on RCC is inconclusive. There are many reasons for this. First, and perhaps the most important, is the lack of an adequate number of studies. This is surprising given the direct link between RCC and VHL mutations, the direct or indirect regulation of EPO expression by VHL and the involvement of HIF. Second, the sample size of the available studies is inadequate to evaluate the prognostic significance of EPO and EPOR expression in RCC. Third, the effects of EPO administration in RCC patients (or in other cancers), either beneficial or adverse, cannot be correlated to the expression status of EPO or EPOR, because the criteria for patient selection were not based on the expression status of either of these molecules, and to date no studies have explored this aspect. More comprehensive studies using human samples are warranted. In particular, further information on the baseline level of EPO and EPOR in RCC would be of value in monitoring the effect of exogenous rhEPO on the progression of RCC.

## Abbreviations

EMT: Epithelial-mesenchymal transition; EPO: Erythropoietin; EPOR: Erythropoietin receptor; HIF: Hypoxia-inducible factor; IL-2: Interleukin-2; PTEC: Proximal tubular epithelial cells; RCC: Renal cell carcinoma; rhEPO: Recombinant human erythropoietin; VEGF: Vascular endothelial growth factor; VHL: von Hipple-Lindau.

## Competing interests

Professor David Johnson is a current recipient of a Queensland Government Health Research Fellowship. He has received consultancy fees, research funds, speaking honoraria and travel sponsorships from Jannsen-Cilag, Amgen, Pfizer and Roche. All other authors verify that they have nothing to disclose.

## Authors’ contributions

CM and GCG contributed to the conception of the idea, literature search and drafting the manuscript. DWJ and DAV contributed to the interpretation of findings, critical evaluation and editing of the manuscript. All authors critically reviewed and accepted the final version of the manuscript.

## Pre-publication history

The pre-publication history for this paper can be accessed here:

http://www.biomedcentral.com/1471-2407/13/14/prepub
